# Phytochemical Evaluation of *Terminalia canescens* DC. Radlk. Extracts with Antibacterial and Antibiotic Potentiation Activities against Selected β-Lactam Drug-Resistant Bacteria

**DOI:** 10.3390/molecules29061385

**Published:** 2024-03-20

**Authors:** Muhammad Jawad Zai, Matthew James Cheesman, Ian Edwin Cock

**Affiliations:** 1 Centre for Planetary Health and Food Security, Griffith University, Brisbane, QLD 4111, Australia; muhammadjawad.zai@griffithuni.edu.au; 2 School of Environment and Science, Griffith University, Brisbane, QLD 4111, Australia; 3 School of Pharmacy and Medical Sciences, Griffith University, Southport, QLD 4222, Australia; m.cheesman@griffith.edu.au

**Keywords:** *Terminalia canescens*, antibiotic-resistance, ESBL, MRSA, flavonoid, tannin

## Abstract

*Terminalia canescens* DC. Radlk. (family: Combretaceae) is native to northern Australia. Species of the genus *Terminalia* are widely used as traditional medicines to treat diverse ailments, including bacterial infections. However, we were unable to find any studies that had examined the antimicrobial activity of *T. canescens*. In this study, *T. canescens* was screened against a panel of bacterial pathogens, including multi-antibiotic-resistant strains. Solvents with different polarities were used to extract different complements of phytochemicals from *T. canescens* leaves. Methanolic and aqueous extracts exhibited substantial antimicrobial activity against various pathogens, including those that are multidrug-resistant strains. When combined with some selected clinical antibiotics, some extracts potentiated the antibacterial inhibitory activity. This study identified two synergistic, eleven additive, eleven non-interactive and eight antagonistic interactions. The toxicities of the plant extracts were examined in the *Artemia franciscana* nauplii assay and were found to be non-toxic, except the aqueous extract, which showed toxicity. Metabolomic liquid chromatography–mass spectrometry (LC-MS) analyses highlighted and identified several flavonoids, including vitexin, quercetin, orientin and kaempferol, as well as the tannins ellagic acid and pyrogallol, which may contribute to the antibacterial activities observed herein. The possible mechanism of action of these extracts was further explored in this study.

## 1. Introduction

Recent increases in bacterial antimicrobial resistance (AMR) have resulted in increased mortality and morbidity globally [1]. Multidrug resistance (MDR) in Gram-negative and Gram-positive bacteria has decreased the effectiveness of existing broad-spectrum antibiotics [1]. The increase in AMR has led to the loss of affordable and effective treatments for infections as well as decreased efficacies of penicillin and oxacillin for treating *Staphylococcal* infections, sulphonamides and ampicillin for urinary tract infections, and penicillin and fluoroquinolones for combating gonorrhea [2]. AMR has serious implications for public health and social and economic implications. It affects both hospital-acquired infections, including methicillin-resistant *Staphylococcus aureus* (MRSA), vancomycin-resistant *S. aureus* and vancomycin-resistant *Enterococci*, and extended-spectrum β-lactamase (ESBL) enzyme producing Gram-negative bacteria. AMR impacts community-acquired infections, including pneumonia, typhoid fever and *Streptococcal* infections. AMR leads to longer hospital stays and increases the cost of treatment, which increases the economic impact on the community [3]. The combination of a heavy disease burden, poor sanitation and the improper use of antibiotics contributes to the development of AMR. Numerous organizations, including the Infectious Disease Society of America (IDSA), the Centers for Disease Control and Prevention (CDC), the World Health Organization (WHO) and the World Economic Forum (WEF) have highlighted antibiotic resistance as a major concern to public health globally [4,5]. Indeed, WHO have proposed a global action plan to fight the problem of AMR [6].

There has been a substantial increase in research efforts aimed at developing novel treatments against multidrug-resistant (MDR) and extensively resistant bacterial species. In recent times, traditional and herbal medicines have attracted considerable attention as potential candidates for new drug discovery targets [7]. The increasing interest in medicinal plants is supported by a mounting body of evidence reporting their antimicrobial properties, and the ability of some plant extracts to potentiate the efficacies of some clinical antibiotics [8]. The genus *Terminalia* consists of approximately 250 species, with a significant number of its species used traditionally to treat diverse ailments. These therapeutic uses include (but are not limited to) diarrhea, headache, gastric ulcers, heart disease, skin disease and bacterial infections [9]. Phytochemical investigations into several *Terminalia* species have identified several phytochemical classes, including tannins, triterpenes and glycoside derivatives, along with flavonoids and other polyphenolic compounds [10]. This variety of phytochemicals may contribute to the medicinal properties of *Terminalia* spp. Despite the previous reports of medicinal properties of multiple *Terminalia* species, the therapeutic potential of *Terminalia canescens* DC. Radlk remains unexplored.

This study assesses the bacterial-growth inhibitory activity of *T. canescens* leaf extracts against a panel of bacterial spp. sensitive to β-lactam antibiotics, as well as antibiotic-resistant strains. The bacteria examined in this study included *K. pneumoniae* and ESBL *K. pneumoniae*, *S. aureus* and MRSA, as well as *E. coli* and ESBL *E. coli*. Our focus was on β-lactam resistance, although the analyzed strains also exhibit resistance to multiple other classes of antibiotics [11]. Furthermore, antimicrobial activity of *T. canescens* extracts was also tested in combination with conventional antibiotics to determine whether plant extracts potentiate the activity of the antibiotics in the combination. The rapid increase in β-lactam resistance pathogens is alarming considering our historical reliance on this class of antibiotics as broad-spectrum antibiotics. Additionally, high performance liquid chromatography–mass spectrometry (LC-MS) analysis of the *T. canescens* extracts was used to identify notable compounds in the extracts. The extracts’ toxicities were evaluated by an *Artemia franciscana* (ALA) nauplii toxicity assay.

## 2. Results

### 2.1. Antimicrobial Susceptibility Studies

**Figure 1:**
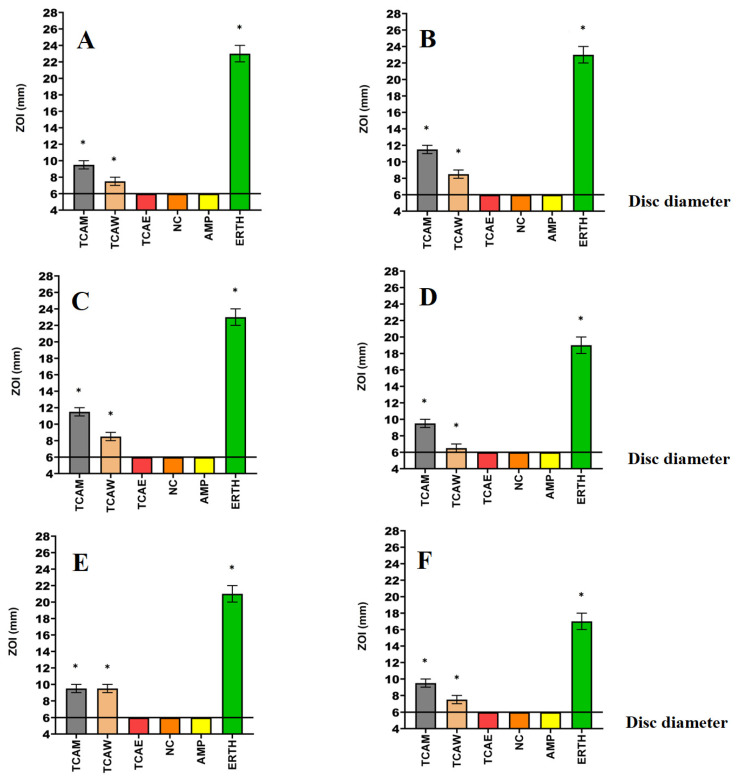
Antimicrobial effects of the *T. canescens* leaf extracts against (**A**) *K. pneumoniae*, (**B**) ESBL *K. pneumoniae*, (**C**) *S. aureus*, (**D**) MRSA, (**E**) *E. coli* and (**F**) ESBL *E. coli*. TCAM = *Terminalia canescens* methanol, TCAW = *Terminalia canescens* water, TCAE = *Terminalia canescens* ethyl acetate extract. Positive controls = ampicillin (AMP; 2 µg) and erythromycin (ERTH; 10 µg). Negative control (NC) = water. Results are expressed as mean zones of inhibition of three independent replicates ± SEM (*n* = 3). * indicates that the results are significantly different to the negative control (*p* < 0.01).

Powdered *T. canescens* leaves were extracted using solvents of varying polarities, then dried and resuspended in 10 mL of 1% DMSO, resulting in concentrations of 30.7, 16.4, and 4.3 mg/mL for the methanolic, water, and ethyl acetate extracts, respectively. The antimicrobial activity of each extract was initially examined in a panel of bacterial species on agar plates by disc diffusion assays (Figure 1) measured as zones of inhibition (ZOIs). These assays were included to provide an approximation of bacterial infections on solid surfaces. Liquid dilution assays (Table 1) were also used to quantify the antibacterial potency of the extracts by determining minimum inhibitory concentrations (MICs). The *T. canescens* methanol (TCAM) and *T. canescens* water (TCAW) extracts both inhibited the growth of all of the antibiotic-sensitive and antibiotic-resistant bacterial strains tested herein. In contrast, the *T. canescens* ethyl acetate extract (TCAE) did not inhibit the growth of any bacterial species in either the disc diffusion assay or liquid dilution assays. Notably, the methanolic extract had similar activities against the antibiotic-sensitive *K. pneumoniae* and the ESBL *K. pneumoniae* strains (MIC = 960 µg/mL).

### 2.2. Calculation of Fractional Inhibitory Concentration

The *T. canescens* leaf extracts were also tested in combination with a panel of conventional antibiotics to evaluate the effects of the extracts on the antibiotics function. Several classes of interactions were observed when the combinations were tested against antibiotic-sensitive and antibiotic-resistant *K. pneumoniae*, *S. aureus* and *E. coli* (Table 2). Notably, two combinations, comprising TCAM in combination with ciprofloxacin against *E. coli,* and TCAW in combination with ciprofloxacin against *K. pneumoniae*, exhibited synergistic effects. Therefore, there would be substantial advantage in using these combinations against these pathogens, rather than using either the extract or antibiotic component alone. Additionally, eleven combinations were additive, eleven combinations were non-interactive, and three combinations were antagonistic. As the antagonistic combinations have decreased efficacy compared to using either component separately, these combinations should be avoided against those bacteria.

### 2.3. Evaluation of Extract and Antibiotic Synergistic Interactions at Different Ratios

**Figure 2:**
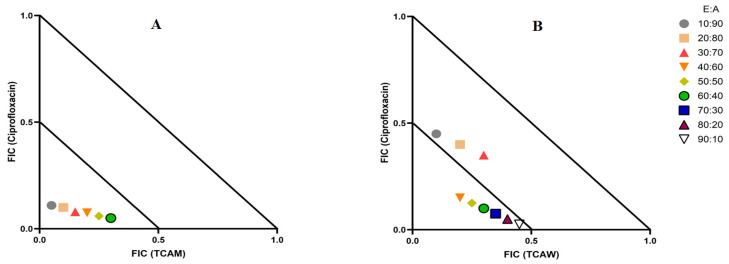
(**A**) Isobologram analysis of the TCAM and ciprofloxacin combination when tested at multiple ratios against *E. coli*; (**B**) Isobologram of ratios of TCAW extract in combination with ciprofloxacin against *K. pneumoniae*. FIC values are displayed as the means of two independent repeats (*n* = 2). Ratio = % extract: % antibiotic. Values below the 0.5/0.5 line represent synergy; the segment between the 0.5/0.5 and 1/1 lines represents additive interactions. Only the synergistic and additive ratios are displayed in these graphs.

Two combinations of extracts and antibiotics were synergistic: TCAM combined with ciprofloxacin and the combination of TCAW and ciprofloxacin (Table 2). Therefore, multiple extract/antibiotic ratios were tested using isobolograms and the fractional inhibitory concentrations were plotted to identify the synergistic ratios. Only the synergistic and additive combinations are displayed in the isobolograms (Figure 2). The TCAM and ciprofloxacin combination exhibited synergy against *E. coli*, although only in ratios consisting of 10–60% extract. In contrast, the TCAW and ciprofloxacin combination produced synergistic interactions against *K. pneumonia,* although only at ratios containing 40–90% extract, while ratios containing 10–30% extract produce additive effects. Ratios that were non-interactive have no additional benefits over the individual components alone and hence were not included in the isobolograms.

### 2.4. Identification of Compounds in the TCAM and TCAW Extracts

**Figure 3:**
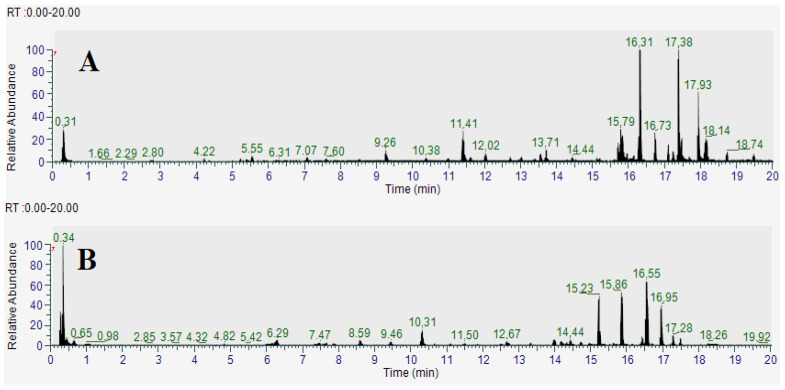
Total compound LC-MS chromatograms in positive ionization mode of (**A**) TCAM (*T. canescens* methanol) and (**B**) TCAW (*Terminalia canescens* water) extracts.

TCAM and TCAW extracts showed the greatest antimicrobial activity in the disc diffusion susceptibility tests, as well as in the liquid dilution assays, and were therefore deemed to be the most promising extracts for phytochemical separation and identification studies. Optimized parameters that were previously developed in our group for high-performance liquid chromatography–mass spectrometry (HPLC-MS) [12] were used to analyze the metabolomic profile of these extracts, with a focus on flavonoid and tannin components. The resulting total compound chromatograms in positive ionization mode for TCAM and TCAW are shown in Figure 3A and Figure 3B, respectively. The analysis identified a variety of compounds in the TCAM and TCAW extracts, of which the flavonoids and tannin components are listed in Table 3.

### 2.5. Quantification of Toxicity

The toxicity of the extracts (across the range 125 to 1000 µg/mL) was screened using *Artemia franciscana* nauplii (ALA) toxicity assays. Extracts tested at concentrations that induced <50% mortality were deemed to be non-toxic at those concentrations. Extract concentrations that produced >50% mortality were diluted until a concentration that induced <50% mortality was identified. The methanol and ethyl acetate extracts were both non-toxic (<50% mortality at 1000 µg/mL). In contrast, the water extract induced >50% toxicity at 1000 µg/mL, and was then further diluted to determine the LC_50_. For the aqueous extract, the LC_50_ was calculated to be 500 µg/mL and hence was classified as nontoxic. In contrast, the positive control (2 mg/mL potassium dichromate) induced 100% mortality, while the negative control (seawater) induced 0% mortality.

## 3. Discussion

Aqueous and methanolic *T. canescens* leaf extracts inhibited the growth of all six bacterial species tested in the disc diffusion and the liquid dilution assay, including the antibiotic-resistant strains. The methanolic extract was generally a substantially more potent inhibitor of bacterial growth. In contrast, the ethyl acetate extract failed to inhibit the activity of any bacterial strain. The difference in the antibacterial activity of these extracts may be due to the specific phytochemicals extracted from *T. canescens* using solvents of different polarities. Polar solvents generally extract more phytochemicals from plants and in greater abundance compared to lower-polarity solvents [13]. These compositional differences may account for the varying levels of antibacterial activity observed between the disc diffusion and the liquid dilution assay. Phytochemicals with lower polarity and/or larger molecular sizes are hindered while diffusing through solid-phase agar, potentially influencing the perceived antimicrobial efficacy of the extracts in disc diffusion assays [13]. Additionally, the volatile extract components may evaporate from the surfaces of agar gel, leading to decreases in their concentration in the assay, and consequently decreases in their apparent effectiveness [14]. Disc diffusion assays are also influenced by the solubility of the extracted compounds in aqueous solutions, with polar compounds diffusing faster than less soluble compounds. Indeed, the low-solubility compounds may remain concentrated around the disc, which may result in underestimating the MIC values of the extracts [15]. The liquid dilution assay is regarded as more sensitive than the disc diffusion assay, as it is less susceptible to the impacts of compound polarity and size.

Interestingly, the *T. canescens* extracts showed similar inhibitory effects against bacterial species that were resistant to bacteria, compared to their susceptible counterparts. This indicates that compounds present in the *T. canescens* extracts are relatively unaffected by the MRSA and ESBL antibiotic-resistance mechanisms. Therefore, *T. canescens* extract components function via mechanisms that are distinct from those targeted by the antibiotics to which these strains are resistant, particularly the β-lactam antibiotics. Alternatively, specific extract constituents may block the bacterial resistance mechanisms. This is promising, as the antibiotic-resistant bacterial strains examined in this study exhibited significantly diminished susceptibilities to various antibiotics of diverse classes, including tetracyclines, aminoglycosides, fluoroquinolones, sulfonamides, macrolides and β-lactams. Furthermore, the efficacies of *T. canescens* extracts against both Gram-negative and Gram-positive bacteria highlights their potential as broad-spectrum antibiotics. To comprehensively assess their antibiotic potential, future studies should screen the *T. canescens* extracts against a more comprehensive panel of pathogenic bacteria, including other MDR strains.

There is substantial recent interest in the use of plant extracts for potentiating clinical antibiotics against diverse bacterial strains [16]. We observed two synergistic, eleven additive, eleven non-interactive, and eight antagonistic interactions in this study. Synergistic combinations possess substantially enhanced antibacterial effectiveness compared to additive interactions, which also amplify antibiotic potency, albeit to a lesser extent than synergistic interactions. Synergy was observed when combinations of TCAM extract and ciprofloxacin were tested against *E. coli*, and for the combination of TCAW extract and ciprofloxacin against *K. pneumoniae*. Ciprofloxacin targets the alpha subunit of DNA gyrase and prevents it from supercoiling the bacterial DNA which prevents the replication DNA [17]. The plant extracts tested in this study may contain components that block the resistance mechanism of ciprofloxacin, although this remains to be verified. Additionally, the TCAM and TCAE extracts were determined to be nontoxic in the ALA toxicity assay, while TCAW was found to be toxic. Notably, brine shrimp assays are sensitive to changes in pH [18], and this may provide erroneous results. Acidic pH can negatively impact the rate of mitochondrial protein synthesis and may become fatal to the development and growth of nauplii [18]. Water extracts higher levels of organic acids and other acidic compounds than do many other solvents, which may account for the higher levels of toxicity noted for the aqueous extract in our study, compared to the methanolic extract. Therefore, it is suggested that future studies should determine the toxicity of *T. canescens* extracts using human dermal fibroblast toxicity assay, which is a more robust toxicity assay, and not influenced by the pH levels. Conversely, non-interactive combinations neither boost nor diminish the antibacterial effects of the extract or antibiotic components, indicating that they are safe for simultaneous use, despite providing no additional benefit over using either component alone. Notably, antagonistic combinations reduce the antibacterial activity of combinations of extracts and antibiotics, and thus should be avoided.

**Figure 4:**
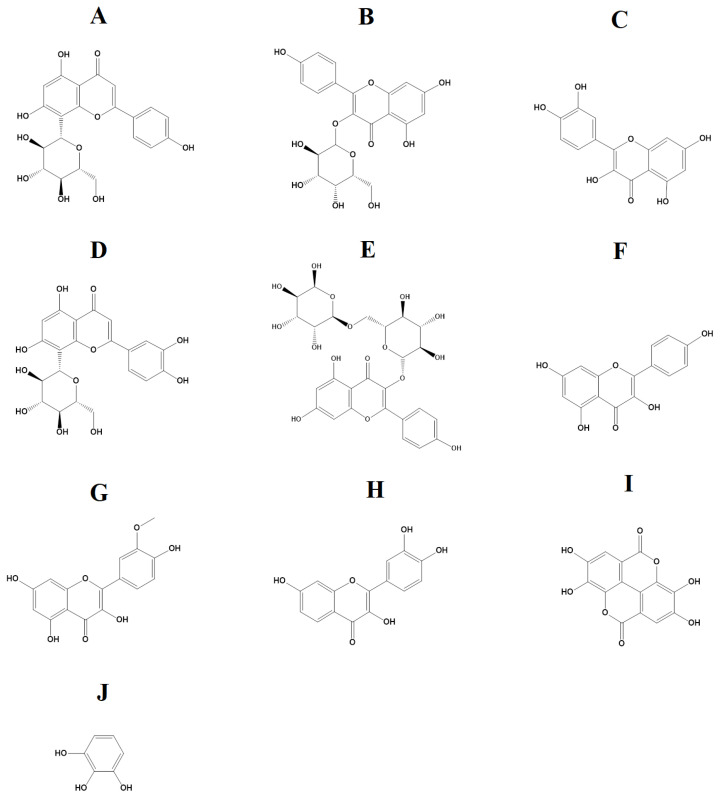
(**A**) Vitexin, (**B**) trifolin, (**C**) quercetin, (**D**) orientin, (**E**) nictoflorin, (**F**) kaempferol, (**G**) isorhamnetin, (**H**) fisetin, (**I**) ellagic acid and (**J**) pyrogallol.

Previous studies in our group have employed LC-MS metabolomics profile analyses to characterize the phytochemical composition of several *Terminalia* species. These analyses aimed to identify and quantify the relative abundances of phyto-constituents within the plant extracts [19]. Several different classes of compounds were identified in *Terminalia* spp. leaf extracts, including the tannins gallic acid, ellagic acid and chebulic acid, and multiple flavonoids. Tannins and flavonoids were also identified in our study in the methanol and aqueous *T. canescens* extracts (Table 3). Specifically, our study detected the flavonoids vitexin (Figure 4A), trifolin (Figure 4B), quercetin (Figure 4C), orientin (Figure 4D), nictoflorin (Figure 4E), kaempferol (Figure 4F), isorhamnetin (Figure 4G) and fisetin (Figure 4H), as well as the tannins ellagic acid (Figure 4I) and pyrogallol (Figure 4J). The antimicrobial properties of flavonoids are well documented against a range of bacterial pathogens [20]. With the increasing prevalence of antibiotic-resistant infections, flavonoids have potential as antibiotic alternatives, as well as in their capacity to overcome or bypass some bacterial resistance mechanisms and therefore potentiate the activity of other compounds.

The therapeutic properties of flavonoids in other species have been well documented. For example, *Tagetes minuta* L., which contains the flavonoid quercetagetin-7-arabinzylgalactoside, is widely used in Argentinean traditional medicine to treat numerous infectious diseases [21]. Studies have also documented the antibacterial activity of flavonoid-rich plant extracts, including *Capsella* spp. [22], *Hypericum* spp. [23] and *Chromolaena* spp. [22]. *Tripleurospermum disciforme* (C.A.Mey.) Sch.Bip. is used to treat of multiple bacterial diseases, and as a disinfectant in Iranian traditional medicine. This species contains relatively high levels of several flavonoids, including quercetin, kaempferol, and their respective glycosides. Quercetin and kaempferol were also detected in the *T. canescens* extracts in our study (Table 3). The antimicrobial properties exhibited by numerous plant-derived flavonoids work through distinct mechanisms compared to conventional drugs, suggesting their potential significance in augmenting antimicrobial therapeutic approaches [24]. Flavonoids (especially catechins) have been reported to have noteworthy antimicrobial properties against both Gram-negative and Gram-positive bacteria. Flavonoids interact with lipid membrane bilayers via two mechanisms [25]. They partition lower polarity compounds into the hydrophobic inner region of the membrane bilayer. Alternatively, flavonoids may induce hydrogen bond formation between the polar head groups of the membrane phospholipids and the flavonoids at the membrane surface. These interactions between phospholipid and flavonoid components induce conformational changes in membrane proteins, including thickness fluctuations, thereby indirectly modulating the function and distribution of membrane proteins.

Tannins also have inhibitory effects on the growth of a diverse range of microorganisms, including fungi, bacteria and yeasts [26]. Tannins function as multidentate ligands, binding proteins through hydrophobic interactions and hydrogen bonds, thereby inhibiting bacterial metabolism [27]. The MICs of tannins against a variety of different pathogens have been reported to be in the range of 61.5–3200 µg/mL [28]. The formation of biofilms is believed to be linked to a substantial proportion of persistent and chronic bacterial infections, with estimates indicating that more than 60% of all bacterial infections result in biofilm development [29]. Extracts that are rich in ellagic acid, which was identified in our study (Table 3), reduce the biofilm activity of *E. coli* and *C. albicans* [30]. In contrast to conventional antibiotics, which generally eliminate bacteria, ellagic acid seems to modify bacterial behaviour by selectively inhibiting biofilm formation, with minimal impact on planktonic bacteria. Compounds with such characteristics may exhibit reduced susceptibility to the development of bacterial resistance, although further studies are needed to confirm this.

The flavonoids and tannins identified in our study may possess intrinsic antimicrobial properties, or alternatively they may potentiate the effects of other *T. canescens* extract components (and some conventional antibiotics). However, additional studies are required to reveal their effects and the antibacterial mechanisms. Future studies should explore these compounds as potential frameworks for new antibiotic therapies, or as potentiating compounds to enhance the efficacy of existing antibiotics. The *T. canescens* extracts tested in our study were effective against both antibiotic-resistant and susceptible strains, a characteristic that is necessary for the development of novel antibiotic drugs. Notably, some extract compounds remained unidentified using LC-MS metabolomic profiling analysis, and these compounds may also contribute to the activity of these extracts. Additionally, lower polarity compounds may not have been detected, and some volatile components may have evaporated during the extraction and/or assay processes. Therefore, further phytochemical studies utilizing different methods (e.g., GC-MS) are required for a complete evaluation of the extracts’ compositions. Importantly, the extracts were non-toxic in the *Artemia nauplii* assay, indicating their safety for antibiotic use. However, testing the extracts against an extensive panel of human cell lines is required to further validate their lack of toxicity and therefore to evaluate their safety for medicinal use.

The studies presented herein quantify the effects of these *T. canescens* extracts against planktonic cells in liquid dilution assays. Notably, many bacteria form biofilms as a protective mechanism and antibiotic extracts and isolated compounds may be substantially less effective against biofilm bacteria than against planktonic bacteria. The solid phase disc diffusion assays presented in this study were included as an approximation of the effects of the extracts against bacteria attached to solid surfaces. However, these effects should be confirmed in future studies using specific biofilm assay models. Additionally, future studies are required to evaluate the antibacterial effects (bactericidal and/or bacteriostatic) of the extract components, and to determine the specific mechanisms.

## 4. Materials and Methods

### 4.1. Materials

All chemicals and reagents used in the studies described herein were AR grade and unless otherwise stated were obtained from Ajax Fine-Chemicals Ltd., Taren Point, Australia. Mueller-Hinton broth and agar were purchased from Oxoid Ltd., Thebarton, Australia. All other chemicals and reagents were acquired from Sigma Aldrich, Bayswater, Australia unless otherwise stated.

### 4.2. Plant Collection and Extraction

*Terminalia canescens* leaves were collected from James Cook University, Cairns by Dr Phurpa Wangchuk and provided for this study. A voucher specimen (GU_TcanJCU_2022) is stored in the School of Environment and Sciences, Griffith University, Australia. A Sunbeam food dehydrator was used to dry the leaves, which were subsequently ground into a fine powder. Methanol, water, or ethyl acetate (50 mL each) were added to individual tubes containing one gram dried and powdered leaf. Extraction of plant material was carried out by maceration for 24 h at room temperature. Whatman No. 54 filter paper was then used to remove particulates from the extracts. A vacuum oven was used to dry the extracts and the mass or dried extract was determined. The dried extracts were reconstituted by adding 100 µL of DMSO to partially dissolve the pellet and increased to 10 mL total volume using sterile deionized water. A syringe-driven filter (0.22 µm; Millipore North Ryde, Australia Pty Ltd., Macquarie Park, Australia) was used to filter the extracts. The extracts were then stored at 4 °C till further use.

### 4.3. Antibacterial Studies

#### 4.3.1. Growth of Bacterial Cultures

Initially, bacterial stock cultures were individually streaked onto Muller–Hinton agar plates and incubated for 24 h at 37 °C. A single bacterial colony was isolated and transferred into Muller–Hinton broth (50 mL) and incubated at 37 °C until the bacterial log growth phase was achieved. The purity of the culture was ensured by re-streaking the culture onto fresh Muller–Hinton agar plates.

#### 4.3.2. Disc Diffusion Assay

Standard methods were followed to examine the antibiotic susceptibility of each bacterial strain by using standard Kirby–Bauer disc diffusion assays [12].

#### 4.3.3. Liquid Microdilution MIC Assay

The MIC values of the plant extract and antibiotics were determined through liquid microdilution assays using standard methods [12].

### 4.4. Determination of Combinational Effects and Optimal Ratios through Isobologram

The interaction effects between the plant extracts and selected conventional antibiotics were studied in 1:1 ratios of the components. The MICs were determined using the same method described in Section 4.3.3. The following formula was then used to calculate the fractional inhibitory concentration (FIC) values:

FIC (E) = (MIC of E in combination with A)/MIC of E alone.

FIC (A) = (MIC of A in combination with E)/MIC of A alone.

∑FIC = FIC (E) + FIC (A).

where E = plant extract; and A = antibiotic. ∑FIC values ≤ 0.5 were termed as synergistic, >0.5–≤1.0 were categorized as additive, values >1.0–≤4.0 were classed as non-interactive, and ∑FIC values > 4.0 were designated as antagonistic.

The combinations which yielded synergistic interactions were subsequently examined at various ratios to determine the ideal ratio(s) at which synergy occurs. The same protocol described in Section 4.3.3. was followed, although a range of ratios of the extract–antibiotic combination were tested. The ratios ranged from 100% antibiotic to 0% extract, with a 10% reduction in increments, and from 0% antibiotics to 100% extract, with a 10% increase in increments. The assay was performed in duplicate, and the resulting data was used for the calculation of FIC values. Isobolograms were plotted and used to identify the ratios that produced synergistic interactions occurring between plant extract (E) and antibiotic (A).

### 4.5. Non-Targeted Head Space LC-MS Conditions for Quantitative Analysis

The identification of different compounds in the extracts was achieved by using non-targeted headspace metabolic profiling. Liquid chromatography–mass spectrometry (LC-MS) analysis was conducted using standard methods previously developed in or laboratory [31]. Putative compound identification was accomplished through molecular annotation against the ChemSpider, CyanoMetDB, mzVault, mzCloud and Global Natural Product Social Molecular Networking (GNPS) databases, as well as via comparisons with published data.

### 4.6. Toxicity Studies

The toxicity of plant extracts was determined by employing *Artemia franciscana* nauplii lethality assays (ALA) using standard methods [12].

## 5. Conclusions

The urgent need to address AMR has led to a substantial increase in the number of studies testing extracts and natural products as sources for novel antibiotic therapies. Our study reports that *T. canescens* has inhibited the activity of MDR and ESBL bacteria as effectively as in the cases of the sensitive strains. This shows that plant extract phytochemicals may have novel and/or unstudied antimicrobial mechanisms. Also, some of the compounds identified herein may contribute to the antimicrobial activities. Future studies are planned to investigate the antibacterial properties of these compounds. Additionally, studies should also further explore the effectiveness of these compounds to potentiate conventional antibiotics by determining the potentiation mechanisms.

## Figures and Tables

**Table 1:** MIC values (µg/mL) of plant extracts and conventional antibiotics against the bacteria tested in this study.

Extract and Antibiotic	MIC (µg/mL)					
	*E. coli*	ESBL *E. coli*	*S. aureus*	MRSA	*K. pneumoniae*	ESBL *K. pneumoniae*
TCAM	1919	1919	960	1919	960	960
TCAW	2050	2050	1025	2050	2050	2050
TCAE	-	-	-	-	-	-
Tetracycline	-	-	1.25	-	-	-
Chloramphenicol	-	-	0.31	-	1.25	1.25
Ciprofloxacin	2.5	-	0.62	2.5	2.5	1.25
Gentamicin	0.039	0.039	0.03	0.03	0.03	0.03
Erythromycin	-	-	1.25	-	2.5	-
Negative control	-	-	-	-	-	-

TCAM = *Terminalia canescens* methanol extract, TCAW = *Terminalia canescens* water extract, and TCAE = *Terminalia canescens* ethyl acetate extract; - indicates no inhibition was observed at any concentration tested. MIC values of triplicate determinations (*n* = 3) are shown and are expressed in units of µg/mL.

**Table 2:** ∑ FIC values for interactions between plant extracts and antibiotics.

Bacteria	Extract	Tetracycline	Chloramphenicol	Ciprofloxacin	Gentamicin	Erythromycin
*E. coli*	TCAM	-	-	0.31	2.13	-
	TCAW	-	-	0.75	2.1	
	TCAE	-	-	-	-	-
ESBL *E. coli*	TCAM	-	-	-	2.72	-
	TCAW	-	-	-	2.66	-
	TCAE	-	-	-	-	-
*S. aureus*	TCAM	*0.63*	2	1.50	5.70	*0.63*
	TCAW	*0.75*	3	2	22	*1.5*
	TCAE	-	-	-	-	-
MRSA	TCAM	-	-	*0.62*	5.45	-
	TCAW	-	-	*0.75*	5.32	-
	TCAE	-	-	-	-	-
*K. pneumoniae*	TCAM	-	1.25	1.12	11.4	*0.56*
	TCAW	-	1	0.37	10.6	*0.75*
	TCAE	-	-	-	-	-
ESBL *K. pneumoniae*	TCAM	-	1.25	-	5.70	-
	TCAW	-	*1*	-	5.33	-
	TCAE	-	-	-	-	-

∑ FIC values of plant extracts in combination with conventional antibiotics against sensitive and resistant strains of *E. coli*, *S. aureus* and *K. pneumoniae*. TCAM = *Terminalia canescens* methanol extract, TCAW = *Terminalia canescens* water extract, TCAE = *Terminalia canescens* ethyl acetate extract; **Synergy = ≤ 0.5**; *Additive = > 0.5–1.0*, Indifferent = >1.0–≤4; Antagonistic = >4.0. FIC values were evaluated in duplicate (*n* = 2). - indicates no inhibition at any concentration tested.

**Table 3:** Qualitative analysis of LC-MS of TCAM and TCAW.

	Retention Time (Min)	Empirical Formula	Molecular Mass	Putative Identification	Relative Abundance (% Total Area)	
					TCAM	TCAW
**Flavonoids**	6.18	C_21_H_20_O_10_	432	Vitexin	1.38	
	7.07	C_21_H_20_O_11_	448	Trifolin	3.82	
	6.31	C_21_H_20_O_12_	464	Quercitin-3β-d-glucoside	0.99	
	6.36	C_27_H_30_O_16_	610	Quercitin 3-*O*-rhamnoside-7 -*O*-glucoside	0.50	
	6.31	C_15_H_10_O_7_	302	Quercetin	0.64	
	5.54	C_21_H_20_O_11_	448	Orientin	7.91	
	7.14	C_27_H_30_O_15_	594	Nictoflorin	0.98	
	7.13	C_15_H_10_O_6_	286	Kaempferol	0.19	
	7.24	C_16_H_12_O_7_	316	Isorhamnetin	0.31	
	8.51	C_22_H_20_O_12_	476	Hispidulin 7-glucuronide	2.53	
	7.07	C_15_H_10_O_6_	286	Fisetin	1.10	
	6.10	C_21_H_18_O_14_	494	8-Hydroxytricetin 7-glucuronide	0.04	
	7.25	C_22_H_22_O_12_	478	5,7-Dihydroxy-2-(4-hydroxy-3-methoxyphenyl)-3-{[3,4,5-trihydroxy-6-(hydroxymethyl)oxan-2-yl]oxy}-4H-chromen-4-one	0.43	
	6.88	C_21_H_20_O_11_	448	4-(3,4-Dihydroxyphenyl)-7-hydroxy-5-{[(2*S*,3*R*,4*S*,5*S*,6*R*)-3,4,5-trihydroxy-6-(hydroxymethyl)oxan-2-yl]oxy}-2*H*-chromen-2-one	3.46	
	6.19	C_15_H_10_O_9_	334	3,5,6,7,2′,3′,4′-Heptahydroxyflavone	0.08	
	5.27	C_20_H_18_O_13_	466	2-(3,4-Dihydroxyphenyl)-3,5,7-trihydroxy-8-{[(2*R*,3*R*,4*S*,5*S*,6*R*)-3,4,5,6-tetrahydroxytetrahydro-2*H*-pyran-2-yl]oxy}-4*H*-chromen-4-one	0.04	
	6.58	C_28_H_24_O_14_	584	2″-*O*-Galloylisovitexin	0.19	
	7.43	C_21_H_18_O_12_	462	(2*S*,3*S*,4*S*,5*R*,6*S*)-6-{[5,7-Dihydroxy-2-(4-hydroxyphenyl)-4-oxo-4*H*-chromen-3-yl]oxy}-3,4,5-trihydroxyoxane-2-carboxylic acid	0.13	
	9	C_15_H_10_O_7_	302	Quercetin		0.18
	6.29	C_21_H_20_O_11_	448	Orientin		9.77
	0.35	C_21_H_20_O_14_	496	Hibiscetin 3-glucoside		0.49
	13.70	C_18_H_14_O_9_	374	Gossypetin 7-methyl ether 8-acetate		0.02
	10.03	C_15_H_10_O_6_	286	Fisetin		0.11
	7.63	C_20_H_22_O_5_	342	Brosimacutin C		0.02
	10.61	C_16_H_12_O_7_	316	2-(3,4-dihydroxyphenyl)-3,5,7-trihydroxy-6-methyl-4*H*-chromen-4-one		0.17
	7.35	C_21_H_20_O_10_	432	1,5-Anhydro-1-[5,7-dihydroxy-3-(4-hydroxyphenyl)-4-oxo-4*H*-chromen-8-yl]hexitol		1.03
	6.95	C_28_H_24_O_15_	600	(2*S*,3*R*,4*R*,5*S*,6*S*)-2-{[2-(3,4-Dihydroxyphenyl)-5,7-dihydroxy-4-oxo-4*H*-chromen-3-yl]oxy}-3,5-dihydroxy-6-methyloxan-4-yl 3,4,5-trihydroxybenzoate		0.01
**Tannins**	6.09	C_14_H_6_O_8_	303	Ellagic acid (Isomer 1)	3.84	
	7.47	C_14_H_6_O_8_	303	Ellagic acid (Isomer 2)		4.06
	1.64	C_6_H_6_O_3_	126	Pyrogallol		2.23

## Data Availability

All data are available from the corresponding author upon reasonable request.
